# Molecular mechanisms of vascular tissue patterning in Arabidopsis thaliana L. roots

**DOI:** 10.18699/VJGB-22-88

**Published:** 2022-12

**Authors:** A.D. Sidorenko, N.A. Omelyanchuk, E.V. Zemlyanskaya

**Affiliations:** Institute of Cytology and Genetics of the Siberian Branch of the Russian Academy of Sciences, Novosibirsk, Russia Novosibirsk State University, Novosibirsk, Russia; Institute of Cytology and Genetics of the Siberian Branch of the Russian Academy of Sciences, Novosibirsk, Russia; Institute of Cytology and Genetics of the Siberian Branch of the Russian Academy of Sciences, Novosibirsk, Russia Novosibirsk State University, Novosibirsk, Russia

**Keywords:** merystem, xylem, phloem, (pro)cambium, plant hormones, auxin, cytokinin, Arabidopsis thaliana, меристема, ксилема, флоэма, (про)камбий, фитогормоны, ауксин, цитокинин, Arabidopsis thaliana

## Abstract

A vascular system in plants is a product of aromorphosis that enabled them to colonize land because it delivers water, mineral and organic compounds to plant organs and provides effective communications between organs and mechanical support. Vascular system development is a common object of fundamental research in plant development biology. In the model plant Arabidopsis thaliana, early stages of vascular tissue formation in the root are a bright example of the self-organization of a bisymmetric (having two planes of symmetry) pattern of hormone distribution, which determines vascular cell fates. In the root, vascular tissue development comprises four stages: (1) specification of progenitor cells for the provascular meristem in early embryonic stages, (2) the growth and patterning of the embryo provascular meristem, (3) postembryonic maintenance of the cell identity in the vascular tissue initials within the root apical meristem, and (4) differentiation of their descendants. Although the anatomical details of A. thaliana root vasculature development have long been known and described in detail, our knowledge of the underlying molecular and genetic mechanisms remains limited. In recent years, several important advances have been made, shedding light on the regulation of the earliest events in provascular cells specification. In this review, we summarize the latest data on the molecular and genetic mechanisms of vascular tissue patterning in A. thaliana root. The first part of the review describes the root vasculature ontogeny, and the second reconstructs the sequence of regulatory events that underlie this histogenesis and determine the development of the progenitors of the vascular initials in the embryo and organization of vascular initials in the seedling root.

## Abstract (ru)

Сосудистая система является результатом ароморфоза, который позволил растениям успешно освоить сушу. За счет нее осуществляется проведение воды, минеральных и органических соединений, обеспечивается эффективное сообщение между органами, а также выполняется функция механической опоры. Процесс формирования сосудистой системы – общепринятый объект фундаментальных исследований в области биологии развития растений. В частности, ранние этапы развития сосудистой системы корня модельного растения Arabidopsis thaliana представляют собой яркий пример самоорганизации бисимметричного (имеющего две плоскости симметрии) паттерна распределения фитогормонов, который направляет детерминацию клеток сосудистой системы. В процессе формирования сосудистой системы корня можно выделить четыре этапа: 1) детерминацию (спецификацию) клеток-предшественников проваскулярной меристемы на ранних стадиях эмбриогенеза; 2) рост и разметку проваскулярной меристемы зародыша; 3) постэмбриональное поддержание инициалей (стволовых клеток) сосудистой системы в апикальной меристеме корня; 4) конечную специализацию (дифференцировку) их дочерних клеток. Анатомические детали развития сосудистой системы A. thaliana давно известны и подробно описаны, однако наши знания о молекулярно-генетических механизмах этого процесса все еще ограничены. В последние годы сделано несколько важных открытий, проливающих свет на регуляцию самых ранних событий, предшествующих дифференцировке клеток сосудистой системы. В настоящем обзоре мы обобщаем данные о молекулярно-генетических механизмах, определяющих направление клеточной дифференцировки в элементы сосудистой системы корня у A. thaliana. Первая часть обзора посвящена описанию гистогенеза сосудистой системы корня. Далее мы реконструируем последовательность регуляторных событий, которые лежат в основе этого гистогенеза и обусловливают развитие предшественников инициалей сосудистой системы у зародыша и организацию инициалей сосудистой системы в корне проростка.

## Introduction

Evolutionary formation of a vascular system in plants was a necessary prerequisite for terrestrial colonization (Lucas et al., 2013). Vasculature provides mechanical support, effective transportation of water, and mineral and organic compounds as well as signal molecules and by this has enabled plants to reach enormous sizes and populate different territories. The vascular system consists of two domains different in their structure and functions. These are xylem that provides water transportation and delivers mineral compounds from the root to above-ground organs; and phloem that conveys organic compounds from photosynthesizing tissues rootward (Evert, Eichhorn, 2006).

**Fig. 1.**
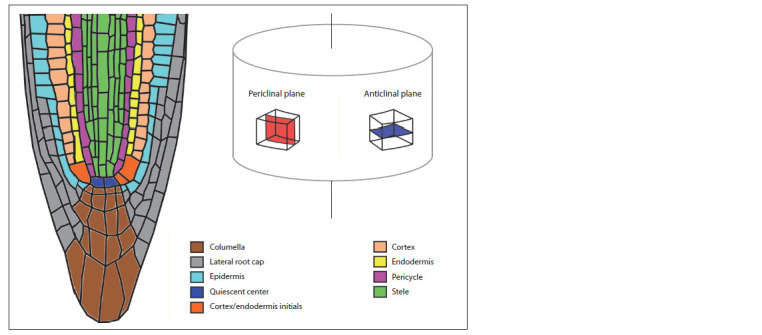
Arabidopsis thaliana root apical meristem The vertical line is the root central axis.

In angiosperms, the mature xylem consists of (1) watertransportation vessels; (2) fibers to provide mechanical support; (3) parenchyma cells (Evert, Eichhorn, 2006). The vessels are the hollow tubes formed by the cells connected in a raw and having perforations in the anticlinal walls and pores in the periclinal walls (Fig. 1). The vessels and fibers are a product of the programmed death of the cells that have formed a lignified secondary cell wall (Courtois-Moreau et al., 2009; Smith et al., 2013; Furuta et al., 2014). Meanwhile, the living cells of parenchyma perform a storage function, participating in vessel lignification and regulating the water transport speed (Ménard, Pesquet, 2015; Růžička et al., 2015).

The phloem, on the other hand, consists of (1) sieve tubes to transport organic substances; (2) companion cells; (3) fibers and sclereids to provide mechanical support, and (4) parenchyma cells (Sjolund, 1997; Evert, Eichhorn, 2006). Unlike the lignified hollow vessels of the xylem, the sieve tubes are a strand of living cells (sieve elements) communicating by sieve fields, anticlinal-wall regions with high numbers of small pores. The sieve elements form a thickened non-lignified secondary cell wall (Heo et al., 2014) and their main feature is the lack most of the organelles including a nucleus, vacuole, rough endoplasmic reticulum, Golgi body, cytoskeleton, ribosomes whose presence could prevent substances transportation. The viability of the sieve elements is maintained by companion cells – the parenchyma cells with large nuclei and mitochondria, directly contacting sieve elements. As for mechanical phloem elements – fibers and sclereids – they differ from each other by the shape of their cells. While the former are strongly elongated and pointed at the ends, the latter are just slightly elongated.

Organization of vascular system is different for different organs in different plant species at different stages of their development (Scarpella, Meijer, 2004; Lucas et al., 2013; Furuta et al., 2014). Nevertheless, the mechanisms determining its development are quite conservative (Li et al., 2010; Seo et al., 2020). Plant cells are not capable of migration, so during morphogenesis, the tissue and organ architecture is formed by regulating the sequence and orientation of cell divisions. In terms of its anatomy, the vascular system development has been described in much detail (Scheres et al., 1994; Evert, Eichhorn, 2006; Miyashima et al., 2013; Furuta et al., 2014; De Rybel et al., 2014b, 2016), however, the molecular and genetic mechanisms responsible for this process are much less known. Our current understanding of these mechanisms is mainly based on the investigation of the model plant Arabidopsis thaliana

In the further sections of this review, we will provide a short description of vascular tissue histogenesis in this plant species and reconstruct the corresponding sequence of regulatory events. We will describe the control of root vascular system development in the embryo and seedling, i. e. the earliest stages of its formation. As for the mechanisms controlling vasculature development at later stages, their description can be found in the recent reviews (see Agustí, Blázquez, 2020; Seo et al., 2020).

Glossary Amphicribral vascular bundle – a vascular bundle in which the phloem surrounds the xylem. Anticlinal – located in a plane perpendicular to the surface of a tissue or organ. Talking about anticlinal cell walls or divisions we will mean an anticlinal plane perpendicular to the central axis of an organ. Anticlinal cell division – cell division in the anticlinal plane that leads to an increase in length. Asymmetric cell division – results in the formation of two daughter cells with different cell fates. Cortex – a cell layer surrounding the endodermis. Diarch vascular bundle – a vascular bundle whose phloem and xylem are located at different radii, wherein two rays of xylem are distinguished. Endodermis – the innermost cell layer surrounding the stele. Hypophysis is the upper cell of the suspensor, which acquires its identity at the 16–32 cell stage; gives rise to the quiescent center (the organizing center of the root apical meristem) and the root cap. Periclinal – located in a plane parallel to the surface of a tissue or organ. Periclinal cell division – cell division in the periclinal plain leading to an increase in the number of cell layers in the radial direction. Pericycle – parenchyma cell layer surrounding conductive tissues and forming the stele outer layer. Primary meristem – formed during embryogenesis. Procambium – indeterminate primary vascular meristem cells located between the xylem plate and phloem poles in the root of Arabidopsis thaliana. Provascular initials – four proembryo cells occurring at the early globular stage to form the entire provascular meristem of the root/hypocotyl, and only it. Provascular root/hypocotyl meristem – primary meristem from which the primary vascular system of these organs differentiates after embryo germination. Root apical meristem – primary root meristem to produce all cells of the root during its post-embryonic growth. Secondary meristem – formed during the postembryonic period. Stele (central cylinder) – primary conductive tissues located in the center of the axial organ, and surrounded by a pericycle. Suspensor – a structure at the base of an embryo that connects it to endosperm and consists of the descendants of a two-celled pro-embryo basal cell. Vascular cambium – secondary vascular meristem to provide root thickening. Xylem plate – a layer of primary xylem cells (or their predetermined precursors) located in the central plane along the root axis

## Root vascular tissue histogenesis

There are primary (produced by the primary meristem) and secondary (produced by the secondary meristem) vascular tissues.

Development of root primary conductive tissues

**Fig. 2.**
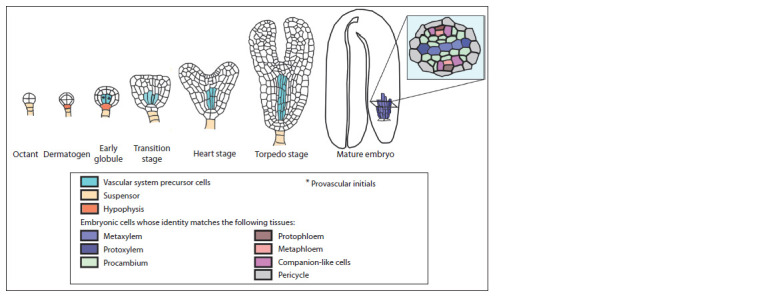
Provascular meristem development in A. thaliana embryo The mature embryo contains predetermined but not differentiated progenitor cells of the future vascular system elements

At the globule stage of A. thaliana embryogenesis the specification of four provascular initials occurs. Provascular initials undergo oriented divisions, finally giving rise to the provascular meristem of the embryonic root and hypocotyl (Fig. 2) (Scheres et al., 1994; Evert, Eichhorn, 2006; Miyashima et al., 2013; Furuta et al., 2014; De Rybel et al., 2014b, 2016). The cells of provascular meristem are not yet differentiated, but the cellular fate of some of them has already been determined – after the embryo germination they give birth either to xylem or to phloem cells. The positions of these predetermined cells in the provascular meristem matches that of the bisymmetric (that is, having two planes of symmetry) diarch organization of the vascular system in the postembryonic root tip: in its transverse – section, there is one layer of xylem precursor cells surrounded on both sides by procambial cells that separate the future xylem from two files of phloem progenitor cells, which lie in a perpendicular plane (Dolan et al., 1993) (Fig. 2, 3, a). This structure is surrounded by pericycle cells that are also derived from provascular initials, so together they form a central cylinder or a stele (see Fig. 1). It is noteworthy that the terminology designating the cells in developing root vascular system is rather blurred (Furuta et al., 2014). In particular, the term ‘procambium’ is applied to address either indeterminate cells of the primary vascular tissue in seedlings (and their progenitors) or the whole embryonic provascular meristem (see Busse, Evert, 1999).

**Fig. 3.**
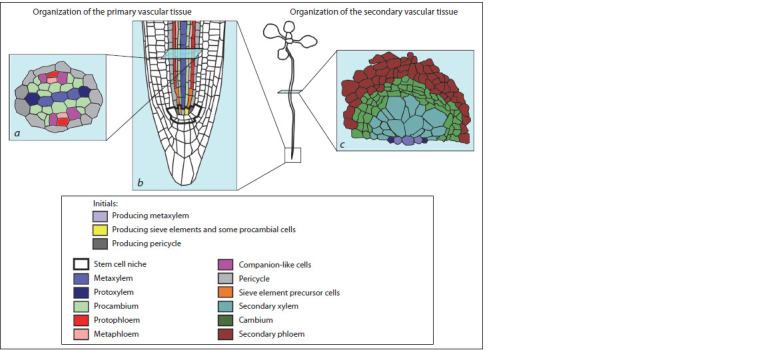
Primary and secondary vascular tissues in postembryonic A. thaliana root. The root-tip stele (a, b) is diarch and comprised of the procambium, primary phloem and xylem surrounded by pericycle. The primary phloem is composed of proto- and metaphloem and of companion-like cells. The primary xylem consists of proto- and metaxylem. In the stem cell niche in longitudinal section (b) two initials producing the procambium/proto-/metaphloem, two initials producing the pericycle, and one producing the metaxylem are visible. During the root secondary growth (c), the cambium produces phloem cells outwards and xylem cells – inwards, so the vascular bundle stops being diarch and becomes amphicribral.

Soon after germination, vascular elements start to differentiate in a hypocotyl stele and cotyledon veins, the provascular meristem of the latter comes from the shoot apical meristem (Miyashima et al., 2013). From the hypocotyl, the process spreads upwards and downwards taking the epicotyl and root, respectively (Busse, Evert, 1999; De Rybel et al., 2014b; Furuta et al., 2014). In A. thaliana, these are the protophloem sieve elements adjacent to the pericycle that differentiate first, and since the cells surrounding them keep elongating, protophloem cells soon die to be functionally replaced by the metaphloem sieve elements placed closer to the center of the stele (Graeff, Hardtke, 2021; Truernit, 2022). Later, the protoxylem vascular elements are formed that are located at the poles of the xylem plate and have annular or spiral thickenings of the secondary cell walls. The last cells to differentiate are the metaxylem cells occupying the central position in the xylem plate and having pitted or reticulate lignin deposits (Růžička et al., 2015).

While the root grows in length, its new cells are produced through anticlinal division of the cells in the apical meristem located at the root tip (Desvoyes et al., 2021). In A. thaliana, the root apical meristem is closed, i. e. different stem cells (initials) can produce not any but strictly limited set of cell types and for each differentiated cell it is easy to trace which stem cell it has originated from (see Fig. 1). Among the stele initials those can be distinguished that give birth to (1) protoxylem; (2) metaxylem; (3) procambium and sieve elements of proto- and metaphloem (in this case, the three cell types are produced through a series of anticlinal and periclinal divisions); (4) only procambial cells; (5) pericycle (see Fig. 3, b) (Mähönen et al., 2000; Rodriguez-Villalon et al., 2015; Truernit, 2022). The mutual arrangement of initials corresponds to the diarch organization of young root vasculature, so the cell identity established in the embryo provascular meristem is maintained in the root apical meristem. Here it is worth mentioning that apart from the proto- and metaxylem, proto- and metaphloem and procambium there are also companion cells. Some authors designate them more srictly as companion-like cells (Truernit, 2022). These cells are adjacent to the sieve elements of proto- and metaphloem and possess a number of morphological and physiological characteristics of companion cells (Stadler et al., 2005; Ross-Elliott et al., 2017; Smetana et al., 2019; Graeff, Hardtke, 2021) but, unlike the latter, they do not share a common initial with the proto- and metaphloem elements in the stem cell niche (Mähönen et al., 2000). The companion-like cells differentiate when the protophloem sieve elements start functioning (Graeff, Hardtke, 2021). In A. thaliana, the xylem and phloem parenchyma, fibers and true companion cells differentiate only during the secondary growth (Růžička et al., 2015; Truernit, 2022).

## Cambium formation

In A. thaliana primary vascular system, periclinal divisions of procambium cells are few, but after differentiation of the primary vascular elements, these cells begin to actively divide periclinally. The periclinal divisions also occur in the pericycle cells adjacent to the xylem plate. As a result, a closed cell ring forms around the xylem to give birth to the vascular cambium (see Fig. 3, c) (Baum et al., 2002; Nieminen et al., 2015; Růžička et al., 2015; Smetana et al., 2019). It is noteworthy that only those procambium and pericycle cells in direct contact with the xylem primary vessels give rise to the vascular cambium, i. e., have the properties of stem cells (Smetana et al., 2019) while the descendants of other proliferating procambial cells differentiate into the phloem.

Thus, the diarch root vasculature transforms into amphicribral one, in which the xylem is surrounded by the phloem with the cambium placed in between (see Fig. 3, c). Through asymmetric division, every initial is capable of producing phloem cells outwards and xylem cells inwards, so the root gets thicker (Smetana et al., 2019). In some species, e. g., in the vast majority of monocots, the cambium is not formed and no secondary growth is initiated. In this case, all procambium cells get differentiated.

## Embryo polarity establishment and the predetermination of provascular initials

The development of a multicellular organism is accompanied by a gradual increase in the limitation of cellular potencies. At the first stage of this process predetermination or specification occurs, in other words, the fate of a totipotent cell is established in terms of the progenitor of what type of cells it will become. Meanwhile, the cell remains undifferentiated and can change its fate under certain conditions. The process of cell identity determination involves the local accumulation of signal molecules, which either activate or suppress the activity the gene networks inherent in specific cell types. In this case, an important role is given to the non-cell-autonomous factors able to move between cells and form gradients (Seo et al., 2020).

Provascular stem cells specification at the early globular stage of embryogenesis is preceded by a series of cell divisions and embryo polarity determination (Lau et al., 2012; De Rybel et al., 2014b). The proper accomplishment of these processes is essential for the vascular tissue to begin its development from the right number of cells placed in the right positions. Plant hormone auxin is a key regulator of embryogenesis, whose heterogeneous distribution provides positional information, which directs embryo development (Weijers, Jürgens, 2005; Smit, Weijers, 2015; Mironova et al., 2017). The main auxin effector in embryogenesis is transcription factor (TF) AUXIN RESPONSE FACTOR 5 (ARF5)/MONOPTEROS (MP) (Smit, Weijers, 2015; Verma et al., 2021) and it is believed that forming the auxin signal-distribution pattern is provided mainly due to feedbacks in regulation of phytohormone biosynthesis, its polar intercellular transport and signaling pathway (Sauer et al., 2006; Möller, Weijers, 2009; Lau et al., 2011; Robert et al., 2015). As a result, at the early stages of embryogenesis, auxin is accumulated in the apical cells to determine the embryo polarity (Wabnik et al., 2013). Starting from the early globular stage (32 cells), its maximum is shifted to the upper cells of the suspensor including the hypophysis that later gives rise the quiescent center of the root apical meristem (Friml et al., 2003; Tanaka et al., 2006).

**Fig. 4.**
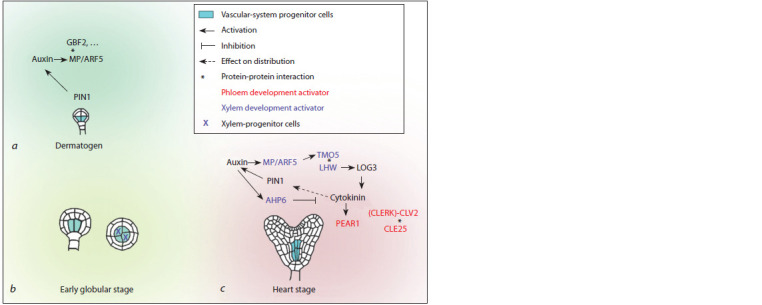
Genetic regulation of provascular meristem development during embryogenesis а, Predetermination of provascular initials. The identity of vascular-tissue progenitors is determined in the four inner cells of the proembryo lower layer at the dermatogen stage. However, anatomically the four initials can only be detected at the early globular stage. b, Xylem progenitor predetermination at the early globular stage; c, formation of the bisymmetric pattern and xylem/phloem progenitor predetermination starting from the heart stage.

Although the four provascular initials are only distinguished at the early globular stage (Scheres et al., 1994), the cellular identity of vascular tissue progenitors is determined in the four inner cells of the lower layer of the proembryo as early as at dermatogen stage (Fig. 4, a) (Smit et al., 2020). Via periclinal division at transferring to the 32-cell stage, they produce outwards the ground tissue progenitors that lose the vascular identity of their maternal cells (see Fig. 4, a, b) (Palovaara et al., 2017; Smit et al., 2020). A necessary condition for provascular- initial specification is ARF5/MP-dependent activation of the auxin signaling pathway, but meeting this condition alone is not enough (Möller et al., 2017; Smit et al., 2020). While particular auxin assistants remain unknown, its is suggested that this role is performed not by a single key regulator but by a multicomponent regulatory network, and TF G-BOX BINDING FACTOR 2 (GBF2) is believed to be one of its members (Smit et al., 2020) (see Fig. 4, a). GBF2 is assumed to modulate ARF5/MP binding to target-gene promoters. It is worth mentioning here that the state, in which vascular system progenitors are uniformly specified is most likely transient with no stable uniform cellular identity.

## Vascular cell predetermination in the provascular meristem

As the oriented divisions of the provascular initials and their descendants continue, the hypocotyl and root vascular systems become patterned through specification of particular cellular types. An important aspect at this stage is setting the boundaries for the cellular domains with different structural and functional identities. By the end of embryogenesis, in the embryo provascular meristem, the cell identity of all elements such as proto- and metaphloem, proto- and metaxylem, companion-like cells and procambium has been determined as evidenced by the data on cell morphology and expression of marker genes (see Fig. 2) (Bonke et al., 2003; Bauby et al., 2007).

In A. thaliana, the bisymmetry of the future root is believed to be predetermined already at the early globular stage by the extended contact between two provascular initials located diagonally relative to each other (see Fig. 4, b). This contact is probably formed due to the inaccurate match of cell division planes in proembryo (at the four-cell stage) and is important for xylem plate formation (De Rybel et al., 2014a). Starting from the early heart stage, auxin begins to be actively transported into such contacting provascular cells from the cotyledon primordia located above them, while in other cells the hormone levels remain low (Bishopp et al., 2011a; Help et al., 2011; De Rybel et al., 2014a). The local increase in auxin concentration is necessary for the specification of xylem progenitor cells (Bishopp et al., 2011a).

At the same time, the cells rich in auxin begin to act as an organizing center for the provascular meristem, coordinating its growth through periclinal divisions and establishment of bisymmetric organization (De Rybel et al., 2014a). Auxin induces the ARF5/MP-dependent expression of TFs TARGET OF MONOPTEROS 5 (TMO5) and TMO5-LIKE1 (T5L1) (Schlereth et al., 2010; De Rybel et al., 2013, 2014b), which, forming heterodimers with the auxin-independent LONESOME HIGHWAY (LHW) TF (De Rybel et al., 2013), activate the expression of cytokinin biosynthesis genes LONELY GUY3 (LOG3) and LOG4 (Kuroha et al., 2009; De Rybel et al., 2014a) (see Fig. 4, c). Simultaneously, auxin blocks cytokinin signal transduction, increasing the expression of gene ARABIDOPSIS HISTIDINE PHOSPHOTRANSFER PROTEIN 6 (AHP6 ) encoding a cytokinin signaling pathway inhibitor (Mähönen et al., 2006; Bishopp et al., 2011a), so a local cytokinin source is formed in xylem progenitors lacking cytokinin signaling

The high cytokinin level, on the one hand, limits auxin efflux from xylem progenitor cells by controlling the localization of auxin transporter PIN-FORMED 1 (PIN1) on the cell membrane (Marhavý et al., 2011; De Rybel et al., 2014a). On the other hand, cytokinin diffuses into neighboring cells following the concentration gradient. In these cells, in the absence of the inhibitor (Cheng, Kieber, 2014), cytokinin activates signaling cascade to stimulate periclinal divisions (Smit, Weijers, 2015). Simultaneously, cytokinin signaling suppresses cell specification into xylem (Mähönen et al., 2006). This mechanism provides for the radial growth of the provascular meristem, which is accompanied by spatial separation of the domains for increased auxin signal (cells obtain xylem identity) and cytokinin signal (pluripotent procambial cells). Its sufficiency for self-organization of the bisymmetric pattern was confirmed using a mathematical model (De Rybel et al., 2014a).

In early embryogenesis, provascular-meristem progenitors begin to express genes encoding peptide hormone CLAVATA 3 (CLV3)/EMBRYO SURROUNDING REGION 25 (CLE25) and mobile TFs of the DNA BINDING WITH ONE FINGER (DOF) family united in the PHLOEM EARLY DOF (PEAR) group marking sieve-element progenitors in the postembryonic period (Miyashima et al., 2019; Ren et al., 2019). CLE25 is expressed starting from a 64-cell embryo stage (Ren et al., 2019). Cytokinin-independent expression of PEAR1 is detected already at a 16-cell stage, and starting from an early heart stage, this gene expression is activated by cytokinin (Miyashima et al., 2019). It is assumed that the CLE25 peptide binding to the CLE-RESISTANT RECEPTOR KINASE (CLERK)-CLV2 receptor together with the PEAR1 TF contribute to the early specification of phloem progenitor cells. However, unlike that for xylem, the mechanism to initiate phloem development in embryogenesis remains unknown.

## Maintaining xylem/phloem-precursor cellular identity in the root apical meristem

Bisymmetric pattern in stele

In the postembryonic period, the stele cells progenitors maintain the bisymmetric pattern established in embryogenesis, so some of the mechanisms regulating the cell dynamics and vascular-system element predetermination in provascular meristem keep functioning even after germination. However, it cannot be said with complete certainty that these mechanisms are identical.

**Fig. 5.**
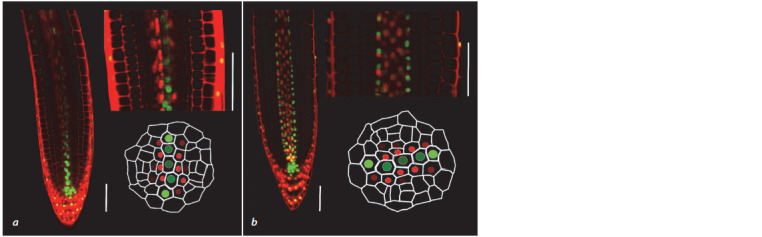
Bisymmetric auxin/cytokinin distribution pattern in A. thaliana root tip stele. a, Xylem plate is located perpendicular to an optical section plane; b, xylem plate is in parallel to an optical section plane. Microimages for the TCSn::ntdTomato-DR5revV2::n3GFP reporter line (Smet et al., 2019) were obtained using a confocal microscope. The cell walls were stained with propidium iodide. GFP (green) and Tomato (red) nuclear signals mark the activity of auxin and cytokinin signaling pathways, respectively. An auxin response is observed in xylem progenitors with the maximum in protoxylem ones, and a cytokinin response – in xylem-adjacent procambial cells, in this way marking the morphofunctional domains of the root tip stele. Scale 50 μm.

**Fig. 6.**
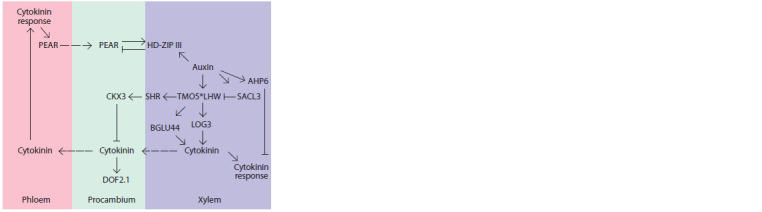
Maintaining the bisymmetric pattern of auxin/cytokinin distribution in A. thaliana root tip stele during the postembryonic period. The star marks a physical interaction between proteins to form a dimer. The dashed arrow indicates mobile regulator movement.

In the apical meristem, auxin-rich xylem progenitors retain the function of an organizing center, carrying out TMO5/ LHW-mediated regulation of cytokinin levels in procambial cells (Fig. 5) (Ohashi-Ito, Bergmann, 2007; Bishopp et al., 2011a; De Rybel et al., 2013; Ohashi-Ito et al., 2013, 2014; Vera-Sirera et al., 2015; Yang et al., 2021). The high content of active cytokinin in xylem cells is maintained by TMO5/ LHW-dependent activation of not only cytokinin biosynthesis genes LOG3 and LOG4 but also of the BGLU44 gene encoding a β-glucosidase enzyme (Fig. 6). Cytokinin response in xylem is blocked by auxin through AHP6 gene expression induction (Bishopp et al., 2011a) as well as through limiting the activity of TMO5/LHW by activating the ACAULIS 5 (ACL5)–SUPPRESSOR OF ACAULIS5 LIKE3 (SACL3) regulatory module blocking the formation of the TMO5/LHW heterodimer by competing with TMO5 for binding to LHW (Katayama et al., 2015; Cai et al., 2016) (see Fig. 6). Meanwhile, in xylem-adjacent procambial cells, the level of the cytokinin diffusing from the xylem is limited by TMO5/ LHWdependent activation of CYTOKININ OXIDASE 3 (CKX3). The activation is mediated by the mobile SHORT ROOT (SHR) TF, encoded by TMO5/LHW target gene. The combined action of multidirectional regulatory modules ensures the stability of the pattern to short-term fluctuations in auxin concentrations in xylem cells, while maintaining its sensitivity to slower/stable changes (Yang et al., 2021). What is interesting is that the SHR gene is important not only for the root radial symmetry but also for the functioning of the quiescent center (Tvorogova et al., 2012).

TMO5/LHW-induced cytokinin activates the transcription of the DOF2.1 TF in the procambial cells surrounding the xylem pole, thus controlling their division (see Fig. 6) (Smet et al., 2019). It is worth noting that, besides xylem cells, it is differentiated phloem that transports the phytohormone and thus can be a source of cytokinin in the root apical meristem (Bishopp et al., 2011b). However, mathematical modeling has demonstrated that phloem cytokinin is not a fundamental source of the positional information for bisymmetric pattern formation (Muraro et al., 2014). At the same time, the high cytokinin content at the phloem poles arranges periclinal divisions of procambium cells through activating the genes of mobile TFs of the DOF family united in the PEAR group including PEAR1, PEAR2, TMO6, DOF6 (Miyashima et al., 2019; Smet et al., 2019). They create a concentration gradient and activate the periclinal divisions of the procambial cells surrounding the phloem pole. HOMEODOMAIN LEU-ZIPPER class-III (HD-ZIP III), TFs whose expression domain is set in the central part of the stele (see below) limit the activity of the PEAR TFs (see Fig. 6), and PEAR1 activates the transcription of the genes belonging to the HD-ZIP III family, forming a negative feedback loop.

Proto- and metaxylem predetermination

**Fig. 7.**
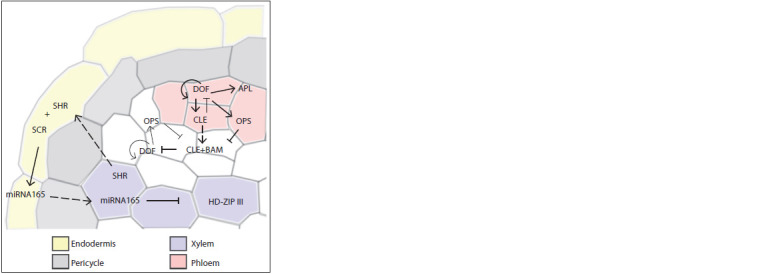
Genetic circuit regulating proto- and metaxylem/phloem cell predetermination in A. thaliana. Separation of the proto- and metaxylem domains is determined by the concentration gradient of the TFs of the HD-ZIP III TF family. The auxin-activated mobile SHR TF diffuses from the xylem to the endodermis and binds to the SCR protein to activate miRNA165 expression. MicroRNAs that degrade HD- ZIP III family TF mRNA form the concentration gradient towards the center and limit HD-ZIP III TF localization to the central domain, thus predetermining metaxylem cells. Phloem predetermination, on the other hand, begins with cytokinin-activated expression of the DOF family TFs. They activate the signal CLE peptides that migrate to neighboring cells, interact with the BAM receptors, and induce DOF degradation to produce a boundary between the future phloem and its neighboring cells. The dashed arrow indicates mobile regulator movement.

As in embryogenesis, auxin is necessary for xylem cells predetermination in the root apical meristem. In proto- and metaxylem predetermination, a key role is given to the SHR and miRNA165/166 mobile regulators (Fig. 7). SHR is produced by xylem cells, from where the TF spreads towards the periphery and, upon reaching the endodermis, activates the SCARECROW (SCR) TF, so they together induce miRNA165/ 166 expression (Carlsbecker et al., 2010; De Rybel et al., 2016). MicroRNAs diffuse into neighboring cells, creating a concentration gradient towards the center of the root. In the stele, miRNA165/166 suppress the expression of the genes encoding the TFs of the HD-ZIP III family, limiting it to the central domain (see Fig. 7). In such a way, the metaxylem cells are predetermined. Whether this mechanism works in embryogenesis remains unknown, but this is a possibility since the PHABULOSA (PHB) TF of the HD-ZIP III family is expressed in the embryo root (Grigg et al., 2009).

Predetermination of phloem elements

The phloem markers expressed in progenitors and induce the tissue development include a number of the DOF family TFs (Miyashima et al., 2019; Roszak et al., 2021); strigolactone signaling pathway suppressors SUPPRESSOR OF MAX2 1-LIKE 3 (SMLX3), SMLX4 and SMLX5 (Wallner et al., 2017); membrane proteins BREVIS RADIX (BRX), OCTOPUS (OPS), OPS-LIKE 2 (OPL2) (Ruiz Sola et al., 2017); phosphatase COTYLEDON VASCULAR PATTERN 2 (CVP2) and its homolog CVP2-LIKE 1 (CVL1) (Rodriguez- Villalon et al., 2015); the ALTERED PHLOEM DEVELOPMENT (APL) TF (Bonke et al., 2003).

The formation of protophloem elements is controlled by shifting the balance towards inducing or suppressing mechanisms with the central link connecting the opposing regulatory modules being phloem-specific TFs of the DOF family (Qian et al., 2022). On the one hand, these TFs induce the expression of phloem development activators, such as APL as well as their own genes, forming a positive feedback loop. On the other hand, DOFs induce the expression of CLE25, CLE26, and CLE45 signaling peptides migrating to neighboring cells where they trigger an inhibitory regulatory module (see Fig. 7). Interacting with the BARELY ANY MERISTEM (BAM) receptors and the CLAVATA3 INSENSITIVE RECEPTOR KINASE (CIK) co-receptors, the CLE peptides induce the degradation of the DOF family TFs, suppressing the formation of protophloem elements. The activity of the CLE peptide receptors can be additionally regulated, e. g., by the MEMBRANE-ASSOCIATED KINASE REGULATOR 5 (MAKR5) (Kang, Hardtke, 2016) or CORYNE (CRN) (Hazak et al., 2017) regulators. The TFs of the DOF family activate the expression of the genes encoding the OPS membrane protein suppressing the BAM-CIK module (Qian et al., 2022). Properly positioned protophloem progenitor cells overcome the inhibitory effect of CLE peptides due to the DOF TF accumulation determined by the positive feedback. Such a balancing mechanism makes it possible to repattern the phloem in case protophloem development has been disrupted (Gujas et al., 2020). Here it should be noted that metaphloem development is probably regulated by other mechanisms and does not depend on that of the protophloem (Graeff, Hardtke, 2021).

During phloem formation, the phloem/procambium stem cell divides anticlinally to produce a daughter procambium and sieve-element progenitor to divide periclinally and form a procambium progenitor and a phloem sieve-element progenitor. The latter undergoes another periclinal division to produce proto- and metaphloem progenitors (Rodriguez- Villalon, 2016). Companion-like cells are another product of asymmetric division, but come from a different initial. These asymmetric cell divisions are controlled by a positional signal, a SHR-protein gradient whose migration into the endodermis activates miRNA165/166 and induces asymmetric divisions producing companion-like cells, while SHR movement into the phloem is necessary for the asymmetric divisions leading to proto- and metaxylem formation (Kim et al., 2020).

## Conclusion

The vascular system of A. thaliana root is set at the earliest stages of embryogenesis. Wherein, the predetermination of provascular initials implies a labile, unstable, and reversible specification based on the physical arrangement of cells in the embryo and influenced by a complex regulatory network of transcription factors. An interesting moment here is that both xylem (e. g., TMO5, T5L1) and phloem (e. g., PEAR1, TMO6, DOF6) markers are jointly expressed by provascular initials in early embryogenesis, but later they are separated into different spatial domains in the provascular meristem and seedling.

In A. thaliana, the vascular system is patterned by the time of embryo maturation. Partially, the gene network that controls this process in embryogenesis continues to maintain the vascular system structure of the growing root of the seedling and later during plant ontogenesis. This is associated with local accumulation of the molecular markers that are stably expressed in progenitor cells of a certain type. However, the factors working both in embryogenesis and during postembryonic development can act at these stages in different ways

Despite the significant progress that has recently been achieved in understanding the molecular and genetic mechanisms regulating vascular system development in plants, many questions remain open, in particular, those related to the existence of parallel regulatory pathways and feedforward loops. This is a good basis for building mathematical models whose analysis helps shed light on the relationship between various regulatory circuits and their functional significance.

## Conflict of interest

The authors declare no conflict of interest.
